# Steady-state photoconductivity and multi-particle interactions in high-mobility organic semiconductors

**DOI:** 10.1038/srep15323

**Published:** 2015-10-19

**Authors:** P. Irkhin, H. Najafov, V. Podzorov

**Affiliations:** 1 Department of Physics and Astronomy, Rutgers University, Piscataway, NJ 08854, USA; 2 Institute for Advanced Materials and Devices for Nanotechnology (IAMDN), Rutgers University, Piscataway, NJ 08854, USA

## Abstract

Fundamental understanding of photocarrier generation, transport and recombination under a steady-state photoexcitation has been an important goal of organic electronics and photonics, since these processes govern such electronic properties of organic semiconductors as, for instance, photoconductivity. Here, we discovered that photoconductivity of a highly ordered organic semiconductor rubrene exhibits several distinct regimes, in which photocurrent as a function of *cw (continuous wave)* excitation intensity is described by a power law with exponents sequentially taking values 1, 1/3 and ¼. We show that in pristine crystals this photocurrent is generated at the very surface of the crystals, while the bulk photocurrent is drastically smaller and follows a different sequence of exponents, 1 and ½. We describe a simple experimental procedure, based on an application of “gauge effect” in high vacuum, that allows to disentangle the surface and bulk contributions to photoconductivity. A model based on singlet exciton fission, triplet fusion and triplet-charge quenching that can describe these non-trivial effects in photoconductivity of highly ordered organic semiconductors is proposed. Observation of these effects in photoconductivity and modeling of the underlying microscopic mechanisms described in this work represent a significant step forward in our understanding of electronic properties of organic semiconductors.

Fundamental understanding of the intrinsic charge carrier transport and optical properties of organic electronic materials can benefit from investigation of organic single-crystal devices with minimized disorder, where the physical behavior is not masked by trapping and scattering. Some of the important processes that govern the operation of organic opto-electronic devices are the polaronic charge transport^1^,^2^ and the dynamics of singlet and triplet excitons^3^,^4^. Rubrene (5,6,11,12-tetraphenylnaphthacene) single crystals represent a convenient test-bed for the investigation of these processes due to several compelling properties, including nearly trap-free charge transport^5^, a high charge carrier mobility of up to ~20 cm^2^V^−1^s^−1^ 5,6,7,8 and high photoconductivity^9^,^10^. In addition, a number of recent studies have suggested that rubrene is a very good triplet material, in which triplet excitons are generated efficiently via singlet fission^9^,^11^,^12^,^13^. Mobile triplet excitons in rubrene have been shown to have a long lifetime of ~100 μs^14^, a large diffusion length of a few μm^9^,^12^, and the ability to undergo fusion to form emissive singlets, thus leading to a delayed photoluminescence (PL) and an anisotropic PL spreading^12^,^14^. These properties make rubrene ideal for fundamental studies of exciton dynamics and photoconductivity. Here, we investigate steady-state photoconductivity in rubrene and report several non-trivial regimes, arising from multi-particle interactions that involve singlet exciton fission, triplet fusion, as well as triplet-charge quenching. Occurrence of some of these processes depends on the probability of one particle to find another, and thus they become notable in systems, where excitons and charge carriers are sufficiently mobile and have long life-times, such as in crystalline organic semiconductors.

We use high-purity vapor grown rubrene single crystals (for details see, e.g.,^15^) with silver or carbon contacts deposited at the top (***a***, ***b***) facet in a coplanar geometry and typically separated by a macroscopic distance *L* = 1–3 mm (that is, much greater than the light penetration length in this experiment) (Fig. 1). The (***a***, ***b***) facet is uniformly illuminated at a normal incidence with a monochromatic light with the penetration length **α**^−1^, where **α** is the absorption coefficient of crystalline rubrene (not to be confused with the power exponent α introduced below). The penetration length **α**^−1^ ~1–10 μm of the light used in this work is much smaller than the typical thickness of our crystals^13^. The photocurrent, *I*_PC_, is measured at a small fixed source-drain voltage, *V*_SD_ = 1–20 V, applied between the contacts. Photoluminescence (PL) is measured simultaneously with photocurrent. We emphasize that geometry of our experiment is such that the distance between the contacts (~mm) is much greater that the light penetration length (~μm), and thus all the layers of the crystal at different depths below the (***a***, ***b***) surface, in which photoexcitation is distributed following a Beer-Lampert exponent, are essentially connected to the contacts in parallel (Fig. 1). In such a “coplanar” geometry with a uniform channel illumination, the voltage applied to each layer is the same (so is the electric field), irrespectively of the depth, photoexcitation level or conductivity of individual layers, meaning that no redistribution of the in-plane electric field should occur when the sample is illuminated. Such a geometry contrasts sharply sandwich type structures illuminated through a semitransparent contact, in which redistribution of the electric field is expected to occur in the material upon photocarrier excitation. Our geometry allows us to define photoconductivity of the sample, *σ*_PC_, as the difference between its conductivity under photoexcitation and the dark conductivity. All our measurements were performed under a small applied electric field, in the range *E*_SD_ = *V*_SD_/*L* = 3–200 V ∙ cm^−1^, at which all the reported effects were found to be independent of this field. A special care has been taken to make sure the crystals do not degrade under photoexcitation in the entire range of excitation power. Most measurements were performed in a clean high vacuum (10^−6^–10^−5^ Torr) with a high-vacuum gauge turned off, unless it was used intentionally, as described below. Optical excitation power (*P*, in nW) refers to the total incident power measured at the surface of the sample. Irradiance, or excitation intensity (*Φ*, in nW/cm^2^) refers to the excitation power per unit of illuminated area of the sample. We also plot our data as a function of the density of absorbed photons (*G*, in cm^−3^s^−1^) defined as the number of photons absorbed per cm^3^ per second at the illuminated surface of the crystal: *G* 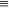 *Φ*/(hν·**α**^−1^), where *h*ν is the photon energy. For further technical details, see Supplementary Information, sec. 1.

Figure 1 shows the typical result of photoconductivity measurements in pristine rubrene performed in a wide range of excitation intensities, covering more than six decades in light power. Again, the pure photocurrent *I*_PC_ (or photoconductivity σ_PC_) is defined as the total current (or conductivity) under illumination minus the dark current (or conductivity) measured at the same small voltage, *V*_SD_, applied between the contacts. The most striking observation is that photoconductivity closely follows a power law, σ_PC_ ∝ *G*^α^, with the exponent α (not to be confused with the absorption coefficient of rubrene **α**) sequentially taking values α = 1, 1/3 and ¼, as the excitation power increases. We reproducibly observe these three distinct regimes in pristine rubrene crystals with a very small standard deviation of 6–12% in the power exponent values (Fig. 1). Note that although non-linearities in photoconductivity were reported in organic semiconductors in the past (see, e.g.,^16^), to the best of our knowledge, the distinct regimes with the power exponents 1/3 and ¼ have not been observed and understood yet.

Before investigating the excitation power dependence of photoconductivity, we will first demonstrate that most of the photocurrent generated in pristine rubrene crystals flows at the very surface of the crystal, perhaps within a ~nm below the (***a***, ***b***) surface, while the bulk contribution is less than ~1% of the total photoconductivity. This very surprising at the first glance observation can be made by carrying out the so-called “*gauge effect*” experiment. Previously, it was observed that high-vacuum gauges generate electrically neutral free radicals that land at the exposed surfaces of molecular crystals or other samples placed in high-vacuum chambers, thus creating surface traps that lead to a noticeable decrease of the charge carrier mobility at the surface and an increase of the threshold voltage in vacuum-gap OFETs, as measured in the dark^17^. Furthermore, it was shown more recently that gauge effect also takes place in graphene^18^. Here, we show that in addition to having an influence on the dark surface transport, high-vacuum gauges also drastically reduce photoconductivity of rubrene crystals (Fig. 2). In this experiment, dark conductivity and photoconductivity of a pristine rubrene crystal (σ_0 _~ 1 nS and σ_PC_ ~ 3.7 nS in Fig. 2, respectively) were monitored in high vacuum (with the gauge off). At *t* = 250 s, a high-vacuum gauge was turned on, which resulted in a very fast and drastic decrease of both σ_0_ and σ_PC_. The very small photoconductivity pulses at *t* > 250 s are produced by the same illumination as the big pulses at *t* < 250 s. The inset in Fig. 2 shows the “gauge effect” on the pure photoconductivity, σ_PC_ (for details on the dark surface conductivity of rubrene see Supplementary Information, sec. 2). It is clear that exposure to the gauge has diminished photoconductivity by a factor of ~60. Given the short-range nature of the gauge effect (see below and sec. 2 of Supplementary Information) and a bulk character of the photoexcitation used in our experiment (light penetration length of several μm is much greater than the lattice constant of rubrene, **α**^−1^ ≫ *c* = 27 Å), this observation implies that most of the photocurrent under illumination is generated and flows at the surface of the crystal.

As discussed in ref. 17, in “gauge effect”, electrically neutral but chemically active free radicals are produced in high-vacuum chambers as a result of a homolytic cleavage of residual hydrocarbons that occurs in high-vacuum gauges or on resistively heated evaporation boats and glowing filaments. Relatively small activation energy and a low threshold temperature of the filament necessary for the gauge effect to occur suggest that it is a homolytic cracking of C-C bond of rather heavy hydrocarbons (residual oil molecules) that is responsible for radical formation. These chemically active species land at the open surfaces of samples placed in high-vacuum chambers and generate traps, thus severely reducing the *surface* conductivity of electronic materials via trapping. The gauge effect appears to be a short-range (surface) phenomenon (Supplementary Information, sec. 2). Indeed, studies have shown that the gauge-effect species are electrically neutral fragments of heavy hydrocarbons, and thus neither long-range Coulomb interactions between these species and charge carriers that might be flowing in the bulk, nor physical permeation of these radicals into the bulk are possible^17^. Additional control experiments show that coating rubrene crystals with a thin protective layer, such as a self-assembled monolayer or a conformal transparent parylene film, with a thickness much smaller than the light penetration length in rubrene, renders photoconductivity absolutely insensitive to high-vacuum gauges (Supplementary Information, Fig. S1). Photoexcitation used in the above experiment (Fig. 2) has a long penetration length **α**^−1^ = 10 μm and is thus generating a Beer-Lampert distribution of singlet excitons essentially stretching into the bulk of the crystal. In conventional (bulk) photoconductivity, photocarriers are created locally, where photons are absorbed, thus leading to samples photoconducting throughout the bulk. Therefore, if a conventional (bulk) photoconductivity took place, a gauge effect would have resulted only in a minor decrease of σ_PC_, because of the short-range nature of the interaction of mobile photocarriers with traps at the surface of the crystal. Contrary to this scenario, we observe a very strong (nearly 100%) reduction of σ_PC_ after the application of the gauge effect, which suggests that photoconductivity in pristine rubrene is predominantly a surface effect. The remaining very small photocurrent, detected after the gauge-effect treatment of the crystal, can now be attributed to a residual bulk photoconductivity. Note that this small residual photocurrent cannot be further reduced even by a prolonged exposure to an operating high-vacuum gauge.

This interesting observation lends a strong support to the model, in which photon absorption in the bulk of the crystal leads to a generation of long-lived excitonic species (for rubrene, triplet excitons generated via singlet fission) capable of reaching the surface of the crystal from the bulk via a long-range diffusion, where they dissociate (with a certain limited quantum efficiency) and generate a surface photocurrent. A possible mechanism of surface dissociation for triplets may involve interactions of triplets with surface defects. However, given the clean, oxide-free surface of pristine rubrene crystals, recently confirmed in surface analytical studies^19^, an intrinsic dissociation due to the lattice discontinuity is more likely. To understand the mechanism of triplet dissociation at the surface more studies are necessary. Irrespectively of the detailed microscopic mechanism of such a dissociation, the gauge effect gives us a powerful and simple experimental tool to selectively trap otherwise mobile carriers only at the surface of the crystal and reversibly switch the sample from a surface to a bulk photoconductor mode, without morphologically or chemically damaging the surface. Reversibility of the gauge effect degradation has been demonstrated in vacuum-gap OFET measurements, when the samples taken out of the vacuum chamber and kept overnight at ambient conditions (in air and under laboratory light) returned to their pristine state^17^. Such a recovery is possible due to the known ability of singlet molecular oxygen (excited via a photosensitized energy transfer from rubrene) to scavenge free radicals (Supplementary Information, sec. 2). The gauge effect allows us to measure photoconductivity and its photoexcitation intensity dependence, σ_PC_(*G*), either at the surface or in the bulk of the same crystal independently. Note that gauge effect should not significantly alter the quantum efficiency of exciton dissociation at the surface of the crystal, because the density of surface traps generated by the gauge is low, 10^9^–10^12^ cm^−2^ (Supplementary Information, sec. 2)^17^. Such trap density is sufficient to immobilize most of the photocarriers generated in our experiment. Indeed, the maximum photocarrier concentration can be estimated by using the maximum *σ*_PC_ from Fig. 3b as *n* = *σ*_PC_/(e*μ*) ≤ 1.25 × 10^10^ cm^−2^ (assuming a carrier mobility *μ* ~10 cm^2^V^−1^s^−1^ at the pristine rubrene/air interface^5^). However, this trap density is much smaller than the density of rubrene molecules comprising the surface of (***a***, ***b***) facet (10^14^ mol./cm^2^). Therefore, a triplet exciton approaching the top surface of the crystals from the bulk would much more likely first encounter a pristine molecular cite, rather than a trap, making any possible interaction of the triplet with such traps statistically insignificant. Thus, although the exact mechanism of triplet dissociation at the free surface of rubrene is unknown, we believe that the extra traps created by the gauge would not affect the triplet dissociation rate averaged over the macroscopic area of the sample’s surface. Instead, they strongly affect the surface photoconductivity via charge carrier trapping. Below we show that *σ*_PC_(*G*) dependencies for the surface and bulk photoconductivity are qualitatively different.

After the relative contributions of the surface and bulk photoconductivities have been established, we focus our attention on the two limiting cases, the surface-dominated and the bulk-dominated photoconductivity, measured in the same crystal in its pristine and gauge-treated states, respectively (Fig. 3b). In addition, photoluminescence power emitted from the (***a***, ***b***)-facet of the crystal has been measured simultaneously with photoconductivity (Fig. 3a). The gauge effect reduces the absolute value of σ_PC_ by 1–2 orders of magnitude, and, more interestingly, leads to a *qualitative* change in the excitation intensity dependence of photoconductivity from a power-law exponent α = 1/3 (surface-dominated) to α = ½ (bulk-dominated). It is interesting that the exact method of introducing surface defects is not important: we have observed the same transition from α = 1/3 to α = ½ after intentional photooxidation of (***a***, ***b***) facets or rubrene, which is known to introduce traps at the surface (Supplementary Information, sec. 3)^20^. Thus, whether the surface traps are introduced by a gauge effect or by any other means is not important, as long as all the surface photocarriers can be immobilized.

Photoconductivity of pristine crystal (black open symbols in Fig. 3b) exhibits the three well-defined regimes characterized by the power-law exponents α = 1, 1/3 and ¼. The bulk photoconductivity of the same crystal, measured after the crystal was exposed to a high-vacuum gauge, has a different behavior with the transitions from α = 1 to ½ and 0.4 (red solid symbols in Fig. 3b). On the lowest intensity end (below 10^14^–10^15^ cm^−3^ s^−1^), the simple linear dependence is always observed. At high excitation powers, a significant increase of PL yield is observed (a “bump” in PL in Fig. 3a), with the inflection point at around 10^20^ cm^−3^ s^−1^, signifying a transition from one linear regime to another one with ~10 times greater slope (note the log scale in Fig. 3a, indicating that this effect in PL is very large). This crossover in PL approximately coincides with the transition from α = 1/3 to ¼ in the surface photoconductivity of pristine crystals. Such a drastic increase of photoluminescence quantum yield thus correlates well with the reduction in the photocarrier generation efficiency, implying that the same new channel responsible for formation of additional emissive singlets is also responsible for the increased photocarrier loss on transition from α = 1/3 to α = 1/4. This correlation and its implications are discussed in more detail below.

The model formulated below rationalizes our experimental observations. The initial photoexcitation in this class of materials results in a short-lived singlet molecular exciton (see, e.g.,^3^). In this work, we simultaneously detect PL emitted by singlets and the excess electrical conductivity (the photoconductivity) that arises when excitons dissociate, thus taking advantage of complementarity of PC and PL. Several recent experimental studies have reported strong evidence that singlet excitons in rubrene efficiently generate triplets via singlet fission, which is supported by the notion that triplet energy in rubrene is almost exactly one half of the singlet energy (see^14^ and refs therein). Singlet fission competes with a direct radiative relaxation of singlets. In addition, two triplet excitons can recombine via triplet fusion to produce an emissive singlet that has the same characteristics as the initially photoexcited singlet. Additional consideration must be added in the case of rubrene (or, other highly ordered small-molecule semiconductors, such as acenes), because the triplet excitons in these material are mobile^9^,^12^, meaning that the probability that they can interact with each other and undergo fusion is high. The radiative recombination of a triplet exciton is quantum-mechanically forbidden, resulting in very long triplet lifetimes of ~100 μs recently measured in rubrene crystals, which is much longer than the lifetime of singlets (a few ns)^14^. This in turn allows for the accumulation of sufficiently large concentrations of triplets under a steady-state excitation, and triplets can start interacting, for instance, with each other or with available free mobile carriers: T_1_ + *n* → S_0_ + *n*. The possibilities of triplet-charge quenching and triplet fusion have been discussed theoretically and experimentally in the context of bulk-heterojunction solar cells and fluorescence measurements^21^,^22^. In addition, because of their long lifetime and diffusion length, triplet excitons are the most feasible candidates for being able to reach dissociation sites (surfaces and interfaces) and contribute to photoconductivity.

We now turn to a model that accounts for the observed power-law dependences of photoconductivity reported in this study. In order to make our physical ideas stand out, we first describe a simplified version of the model by considering only the essential (dominant) processes that we believe are governing the behavior in each regime. We then present a comprehensive model that explicitly lists all the possible processes in a set of differential rate equations. In this full model, individual regimes and power exponents can be derived by assuming a certain single major process that dominates the decay rates in each regime and neglecting the other less significant processes for simplicity. Such an approximation allows to analytically solve the rate equations in many cases and obtain σ_PC_(*G*) dependence explicitly.

In the limit of low illumination intensity, when photoexcitation density *G* is small, and the densities of photogenerated singlet excitons (*S*), triplet excitons (*T*) and photocarriers (*n* 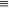 *n*_e_ = *n*_h_) are diluted, we can neglect interactions between these species, because they rarely encounter each other. In this regime, the singlet and triplet densities are proportional to the photoexcitation density (*S*, *T* ∝ *G*), and the rate equation for the charge carrier density *n* is: d*n*/d*t* = κ_1_·*G* − *n*/τ_tr_, where the first term on the right-hand side is the photocarrier generation rate with a quantum efficiency κ_1_, and the second term is the dominant channel of carrier decay—charge trapping on deep traps (τ_tr_ is the carrier lifetime: an average time a charge carrier diffuses before encountering a deep trap and getting immobilized). In a steady state (d*n*/d*t* = 0), this results is a linear dependence of the photocarrier density (and thus photoconductivity) on light intensity: *n* = κ_1_τ_tr_·*G* (the case of α = 1), fulfilled both in the bulk and surface modes of photoconductivity (with different κ_1_ and τ_tr_ parameters in each mode). As *G* increases, in the bulk photoconductivity mode (gauge effect treated crystals), we observe a conventional well-understood bimolecular (*e-h*) recombination regime (α = 1/2), described by the rate equation: d*n*/d*t* = κ_1_·*G* − γ_n_·*n*^2^, where γ_n_ is a bimolecular recombination coefficient for electrons and holes in the bulk, resulting in a square-root dependence of the steady-state photocarrier density *n* = (κ_1_/γ_n_)^1/2^·*G*^1/2^ (the case of α = 1/2). Such a bimolecular photocarrier recombination is dominant at sufficiently high excitation intensities and is very commonly observed in bulk organic and inorganic photoconductors (see, e.g.,^16^).

It is more challenging to understand the α = 1/3 behavior observed in pristine rubrene crystals (that is, in the regime of surface photoconductivity). In order to do so we recall that the photocurrent in pristine crystals is generated almost entirely at the surface of the crystals by virtue of mobile triplet excitons diffusing to and dissociating at the surface of the crystal into mobile charges with a certain quantum efficiency β. We also note that at sufficiently high photoexcitation intensity, densities of mobile carriers and triplet excitons accumulated and coexisting in the near surface region of the crystal could be relatively high, which is due to the long lifetimes of these species. In addition, charge carriers and triplets in rubrene are mobile, and thus the probability of a triplet-charge quenching interaction can be significant. The α = 1/3 can then be derived by assuming that the dominant mechanism of triplet decay in this regime is the triplet-charge quenching, occurring with a rate proportional to the product of the densities of these species (*n*·*T*). Thus, for the triplet density *T* in this regime we have: d*T*/d*t* = κ_2_·*G* − γ_a_·*Tn*, where κ_2_ is a quantum efficiency of triplet generation via singlet fission, and γ_a_ is the coefficient of triplet-charge quenching, leading to a steady-state triplet density *T* = (κ_2_/γ_a_)·(*G*/*n*). We also remember that since surface photoconductivity in pristine crystals originates from triplet excitons, the photocarrier generation rate should be proportional to the triplet density, while the dominant channel of photocarrier decay is still assigned to a bimolecular *e-h* recombination: d*n*/d*t* = β·*T* − γ_n_·*n*^2^. This leads to a steady-state photocarrier density 
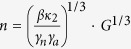
, that is, to the α = 1/3 regime. Finally, the α = ¼ regime can be derived assuming that at even higher excitation density, instead of a triplet-charge quenching, the dominant channel of triplet decay is a radiative triplet-triplet annihilation (triplet fusion) that occurs with a probability proportional to the square of the triplet density (*T*^2^). Thus, in this case we have the following rate equations for triplet and photocarrier populations: d*T*/d*t* = κ_2_·*G* − γ_T_·*T*^2^, where γ_T_ is the coefficient of triplet fusion, and d*n*/d*t* = β·*T* − γ_n_·*n*^2^, resulting in a steady-state photocarrier concentration, *n* = 
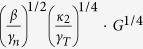
 that corresponds to the α = ¼ regime. We note that the set of parameters κ_1_, κ_2_, γ_a_, γ_n_, γ_T_, τ_tr_ and β may be different in the bulk and surface modes. However, these coefficients do not affect our extraction of the power exponents α.

We now write down the full set of rate equations, containing all the possible processes (at first, without neglecting any), to describe the dynamics of the charge carrier density, and the densities of singlet and triplet excitons, in the presence of singlet-to-triplet and triplet-to-singlet conversions occurring via fission and fusion, as well as triplet-carrier quenching:


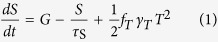



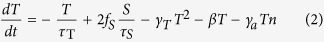



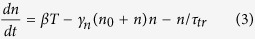


Again, here, *G* is a singlet exciton generation term via light absorption, *n*_0_ is the dark carrier concentration (corresponding to the built-in surface conduction channel existing in pristine rubrene in the dark^17^), *f*_S_ is the probability that singlet decay results in fission into two triples, *f*_T_ is the probability that triplet-triplet collision results in the creation of a singlet via fusion, *τ*_S_ and *τ*_T_ are the singlet and triplet exciton lifetimes, respectively, γ_T_ is a bimolecular recombination coefficient for triplets, and γ_n_ is a bimolecular recombination coefficient for charge carriers. Factors 2 and ½ take into account the fact that fission of one singlet results in two triplets, and vice versa. The term proportional to *T*·*n* that appears on the right hand side of eq. 2 allows for annihilation of triplet excitons via collision with charge carriers, with the corresponding triplet-charge quenching coefficient γ_a_.

We again take into account the conclusion experimentally reached in this work that most of the photocurrent in pristine rubrene crystals is produced via dissociation of triplets at the surface of the crystal. Such dissociation, occurring with a probability *β*, results in a population of photocarriers, *n*, responsible for photoconductivity σ_PC_ = e*nμ*, where e is the elementary charge, and *μ* is the charge carrier mobility. These charge carriers have a lifetime much longer than that of singlet or triplet excitons. As always, in a dynamic equilibrium corresponding to *cw* excitation conditions in our experiment, the steady-state densities of all the involved species are constant: d*S*/d*t* = d*T*/d*t* = d*n*/d*t* = 0.

In the limit of weak photoexcitation, the terms in eqs. 1, 2, 3 corresponding to multi-particle interactions can be neglected (*T *^2^, *T*·*n* ≈ 0) because of the low densities of these species, the density of photogenerated carriers is low (*n*  *n*_0_), and the concentrations of singlets and triplets are proportional to photoexcitation intensity: *S*, *T* ∝ *G* (from eqs. 1, 2), which corresponds to the linear regime *n* ∝ *G* (from eq. 3) in the surface and bulk photoconductivity (α = 1). Note that in this regime, there are two channels of carrier decay in eq. 3: the photocarrier recombination with “dark” carriers (-γ_n_*n*_0_*n*) and the photocarrier trapping on deep traps (-*n*/τ_tr_), both of which can lead to a linear regime. Although we cannot discriminate between these two decay channels, it is clear that in the mode of bulk photoconductivity (gauge effect treated crystals), the linear regime is due to photocarrier trapping, because there are no “dark” carriers in the bulk of ultra-pure undoped rubrene crystals (*n*_0_ = 0).

At somewhat higher excitation intensity, when triplet-triplet and triplet-charge terms are still very small and the generation of singlet and triplet excitons is still linear (*S*, *T* ∝ *G*, from eqs. 1, 2), the carrier concentration can be sufficiently high (*n*    *n*_0_) for a bimolecular *e-h* recombination to dominate, resulting in a *n* ∝ *G*^1/2^ dependence for the bulk photoconductivity (eq. 3), which is a typical result in a variety of bulk photoconductors. The crucial difference with rubrene is that we only observe this regime in the bulk, after the surface photoconductivity is completely suppressed by subjecting the crystal to the gauge effect (Fig. 3b).

The reason bimolecular recombination (α = 1/2 regime) is not observed in pristine crystals is that in the case of a surface-dominated photoconductivity, the concentration of mobile photocarriers and triplet excitons accumulated at and near the surface can get much higher and increase much faster with *G* than that in the bulk, and interactions between triplet excitons and charge carriers become dominant even at low to moderate photoexcitation intensities. In this regime, triplet fusion is still negligible (*T*^2^ ~0, *S* ∝ *G* in eq. 1), and there are only two significant terms in eq. 2: the triplet generation via singlet fission (2f_S_S/τ_S_) and the triplet-charge quenching (γ_a_*T*·*n*), leading to the following relationship between the concentrations of triplets and photocarriers near the surface: *T*·*n* ∝ *G*. Thus, in this regime, we get for the photocarrier density from eq. 3: *n* ∝ *G*^1/3^. It must be noted that although a regime with α = 1/3 can in principle stem from 3^rd^ order (three particle) Auger recombination processes, such processes in rubrene are unlikely, as higher order effects (*ee-h* or *e-hh*) usually occur in indirect gap inorganic semiconductors, where radiative recombination is prohibited, and they require much greater excitation densities (Supplementary Information, sec. 4). In addition, the sequence of exponents occurring with increasing *G* is important: as shown in Fig. S3, in indirect semiconductor *Si*, it is α = 1 (trap dominated), ½ (Shockley-Reed-Hall trap assisted *e-h* recombination) and only then 1/3 (Auger *ee-h* or *e-hh* recombination), while in rubrene, the sequence is α = 1 (trap dominated), 1/3 (*T*·*n* quenching) and then ¼ (*T*·*T* triplet fusion).

At the highest photoexcitation densities (α = ¼ for the surface and 0.4 for bulk-dominated photoconductivity), triplet fusion represented by *T*^2^ term in eqs. 1 and 2 kicks in, strongly competing with all other triplet decay channels in eq. 2, thus leading to *T* ∝ *S*^1/2^ (from eq. 2) and *n* ∝ *G*^1/4^ (from eq. 3) dependences. Note that according to eq. 1 the population of singlets *S* remains linear with *G* even in this case, although with a much greater slope. Indeed, the solution of eq. 1 without and with the triplet fusion term gives *S* = *τ*_S_*G* and S = *τ*_S_*G*/(1 − *f*_S_*  f*_T_), respectively. This indicates that *PL*, which is proportional to the population of singlets *S*, has a linear intensity dependence in both regimes, but the slope increases by a factor of 1/(1 − *f*_S_*   f*_T_) when triplet fusion becomes dominant. This is exactly a kind of behavior we systematically observe in our measurements: a significant (a factor of ~10) increase in photoluminescence yield occurs at around 10^20^ cm^−3^ s^−1^ (Fig. 3a). This suggests that the product *f*_S_*  f*_T_ ≈ 0.9, and thus the probabilities of singlet fission and triplet fusion individually must be very high (  *f*_S_ ≥ 0.9 and *f*_T_ ≥ 0.9), approaching ~100%. This shows that in rubrene both fission and fusion processes are extremely efficient. Note that *f*_S_ and *f*_T_ rates are the probabilities for individual excitons to undergo fission or fusion, respectively. The remaining ~10% of the probability in this excitation regime corresponds to other (non-dominant) channels of exciton decay, such as a radiative recombination for singlets, triplet-charge quenching and triplet dissociation. All these processes are self-consistently accounted for in the system of equations (1, 2, 3). Recent time resolved and steady-state spectroscopic measurements provide more information on exciton fission, fusion and diffusion in rubrene^14^,^23^,^24^,^25^, as well as other crystalline organic semiconductors^26^,^27^.

Finally, it must be noted that in light of the new knowledge of non-linear photoconductivity power dependence observed here, the estimate for the exciton diffusion length, *L*_EX_, in rubrene previously reported by Najafov *et al.*^9^ must be adjusted. The refined procedure should incorporate the 1/3 power dependence in the modeling of the photocurrent modulations, σ_PC_(λ, θ), with excitation polarization angle *θ* and wavelength λ, and it would thus result in somewhat different exciton diffusion lengths. The detailed calculations show that *L*_EX_ remains well above a micrometer (*L*_EX_ > 1 μm) for typical photocurrent oscillations observed in high-quality pristine rubrene crystals (see Supplementary Information, sec. 5, for the refined model).

To summarize, we have shown that contrary to the common expectation of a linear photo-response, highly-ordered organic semiconductors can exhibit a non-linear excitation density dependence of photoconductivity in steady-state measurements. We have devised a simple, yet powerful, experimental method for separating bulk from surface photoconductivity, based on gauge effect. It allowed us for the first time to observe that surface photoconductivity in pristine triplet organic semiconductors, such as rubrene, follows a power law with exponents 1, 1/3 and ¼, while bulk photoconductivity follows the more conventional exponents 1 and ½. We have developed a model based on exciton fission and fusion, as well as triplet-charge quenching, that describes this non-linear behavior. The phenomenological and fundamental understanding of strong nonlinearities in photoconductivity provided by this work is important for gaining deeper insights into the physics of excitons and charge carriers in organic semiconductors and utilization of these materials in emerging photonic and electronic applications.

## Additional Information

**How to cite this article**: Irkhin, P. *et al.* Steady-state photoconductivity and multi particle interactions in high-mobility organic semiconductors.. *Sci. Rep.* **5**, 15323; doi: 10.1038/srep15323 (2015).

## Supplementary Material

Supplementary Information

## Figures and Tables

**Figure 1:**
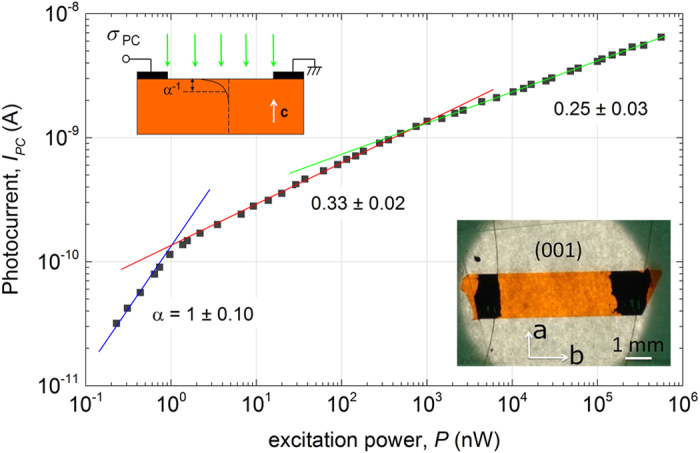
Typical dependence of the steady-state photocurrent on the excitation power in pristine rubrene crystals. Conditions: λ = 500 nm, normal incidence at (*****a*, ***b***) facet, *V*_SD_ = 1 V, excitation intensity is varied over 6 orders of magnitude. Inset on the left: a side-view geometry of the experiment. Inset on the right: a top-view photograph of the typical sample. Photoconductivity follows a power law, σ_PC_(*G*) ∝ *G*^α^, with the three distinct regimes corresponding to the power exponents α = 1, 1/3 and ¼ (note double-log scale). The values of α indicated on the plot for each region correspond to an average power exponent and its standard deviation calculated from five independent measurements of different pristine rubrene single crystals.

**Figure 2:**
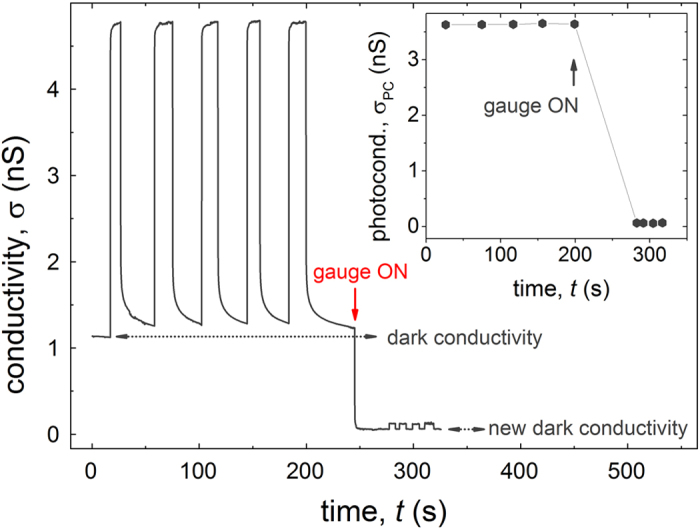
Effect of a high-vacuum gauge on photoconductivity of pristine rubrene. Total conductivity, σ(*t*), is monitored in high vacuum with the high-vacuum gauge off (*t* < 250 s), and after the gauge was turned on (*t* > 250 s). The pulses in σ correspond to a photoexcitation with square light pulses (λ = 532 nm, normal incidence to (***a***, ***b***) facet, *P* = 8 μW, pulse duration 10–20 s, *V*_SD_ = 20 V). The same photoexcitation pulses were used before and after the gauge was turned on. The inset shows the pure photoconductivity, σ_PC_ (with the dark background subtracted), obtained from each pulse. It is clear that the operating high-vacuum gauge diminishes photoconductivity by nearly 100%.

**Figure 3:**
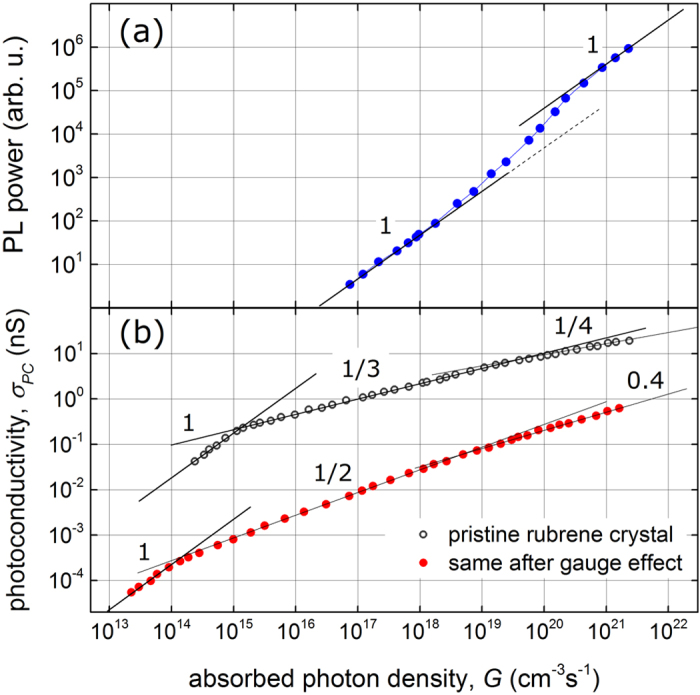
Photoluminescence (a) and photoconductivity (b) simultaneously measured in rubrene crystals as a function of excitation density. Photoconductivity is shown for pristine crystal (black open circles), and for the same crystal after it has been exposed to a high-vacuum gauge (red solid circles). Thin solid lines are power-law fits, σ_PC_ ∝ *G*^α^, with the exponent α indicated next to each line (note the double-log scale). PL was similar before and after applying the gauge because of the bulk nature of singlet exciton generation and emission, as opposed to photoconductivity that has a surface character.
