# Supplementary material for: Steady-state photoconductivity and multi-particle interactions in high-mobility organic semiconductors
Source: Sci Rep. 2015 Oct 19;5:15323. doi: 10.1038/srep15323 (PMC4609983; doi:10.1038/srep15323)
Supplement: Supplementary Information [file srep15323-s1.pdf]

Supplementary Information

Steady-state photoconductivity and multi-particle interactions in high-mobility organic semiconductors.

(July 24, 2015)

P. Irkhin[1], H. Najafov[1] and V. Podzorov[1,2]

[1] Department of Physics and Astronomy, [2] Institute for Advanced Materials and Devices for Nanotechnology (*IAMDN*), Rutgers University, Piscataway, NJ 08854, USA

[*]Corresponding author e-mail address: podzorov@physics.rutgers.edu

1. Experimental methods: sample preparation, measurements and reproducibility.

In this work, we use high-purity, pristine rubrene single crystals grown using an optimized physical vapor transport method (for details see, e.g., [[1]]). Electrical contacts were prepared at the (***a***,***b***) facet of the crystal either by thermally evaporating silver through a shadow mask in high vacuum or by depositing colloidal graphite from an aqueous solution. The contacts are formed in a coplanar configuration (on top of the (***a***,***b***) facet of the crystal) with inter-contact separation ranging from 25 μm to ~ 3 mm. In the typical geometry of our measurements, the (***a***,***b***) facet of the crystal is uniformly illuminated at a normal incidence with a monochromatic light, and photoconductivity, σ_{PC}, is measured at the (***a***,***b***) facet at room temperature by using Keithley K2400 source-meters and K6512 electrometers. All measurements are carried out in small applied electric fields, below 100 $V\cdot cm^{-1}$. Photocurrent (I_{PC}) and photoluminescence (*PL*) are excited with a *cw* (continuous wave) laser emission with a photon energy of 2.3 eV (532 nm) or 2.6 eV (473 nm) in the highly-absorbing energy range of rubrene [[2]], or by monochromatic light produced by a halogen lamp emission sent through a narrow band filter. *PL* generated in the crystal is collected and imaged onto an optical fiber coupled to the Ocean Optics USB2000 spectrometer. A special care has been taken to make sure the crystals do not degrade under photoexcitation during the entire set of measurements and in the entire range of excitation intensities. We used longpass filters with appropriate edge wavelengths to remove the excitation light from the detected *PL* signal. Excitation density was controlled by a set of neutral density filters, while the distribution of light at the sample's surface between the contacts remained constant and very uniform. Most measurements reported here were performed in a clean high vacuum (10^{-5} Torr) with a high-vacuum gauge turned off, unless it was used intentionally, as described in the main text.

It must be noted that at very high excitation intensities, detrimental effects due to local heating, material damage and photooxidation, leading to irreversible changes in the properties of the crystals, may occur. Thus, we took extra precautions to avoid sample degradation under illumination by consistently checking the reproducibility of all the reported data. All the dependencies presented in this work were confirmed to be reproducible, hysteresis free and independent of the measurement history.

2. On the nature of dark conductivity, photoconductivity and gauge effect in rubrene.

The appreciable dark conductivity typically observed at the free (***a***,***b***) surface of pristine rubrene crystals (the so-called *built-in conduction channel*) has been originally attributed to the presence of a monolayer of rubrene endo-peroxide at the surface [3]. This assignment has been prompted by the fact that rubrene molecules in solutions are known to readily photooxidize, leading to rapid bleaching of liquid samples [4]. However, recent detailed studies of crystalline rubrene by the methods of surface analysis, including X-ray photoelectron and secondary ion mass spectroscopies and atomic force microscopy (XPS, SIMS and AFM), have conclusively shown that (***a***,***b***) facets of pristine rubrene crystals are almost free from oxygen [5]. This appears to be due to the tight packing of rubrene molecules in the crystal lattice that makes the surface very resilient against oxidation. Indeed, rubrene single crystals stored in air for as long as a few years (mostly in the dark, but occasionally handled under ambient or microscope illumination) have a surface concentration of atomic oxygen as low as 1-3 % of what would be a monolayer of rubrene fully oxidized to endo-peroxide [5]. Thus, the built-in surface conduction channel in pristine rubrene crystals does not appear to be formed as a result of surface oxidation, but rather occurs via other mechanisms that could be related, for example, to a band bending at the free crystal surface or small relaxation of the surface structure [6]. Similar mechanisms might also be responsible for the surface dissociation of triplet excitons diffusing from the bulk and generating a substantial surface photocurrent [7].

Irrespectively of the exact microscopic mechanism of the dark and photo-conductivity of rubrene, the gauge effect described in the main text gives us a powerful tool to selectively "turn off" the surface and switch the sample from a surface to a bulk (photo)conductor, without morphologically or chemically damaging the surface.

There are several strong arguments in favor of a short-range nature of the gauge effect:

(1). It was concluded that the species generated by high-vacuum gauges are electrically neutral, because the gauge effect degradation appears to be insensitive to various combinations of potentials applied to electric grids inserted between the gauge and the sample in an attempt to prevent the species from reaching the sample [8]. Thus, these charge-neutral species landing at the crystal's surface cannot exert any long-range Coulomb force on the charge carriers that might be flowing in the bulk.

(2). It was also concluded that the species generated by high-vacuum gauges and heated filaments consist of fragments of rather large hydrocarbons (residual molecules of pump oil), and thus they have no chance of physically permeating into the tight lattice of rubrene single crystals below the surface. This assessment was based on the measurement of activation energy of the gauge effect rate as a function of the temperature of glowing filament [8]. This process turns out to have a small activation energy, E_a = 2.5 eV (240 kJ/mol), and a relatively low filament threshold temperature, ~ 950 ^oC, when the gauge effect onset occurs. It is known from the surface science studies that the only reaction possible at such a low energy and temperature is a homolytic cracking of C-C bonds of heavy hydrocarbons (see, e.g., [9], [10], [11]). Bond cracking and dissociation of lighter molecular species present in high vacuum (for instance, residual diatomic or triatomic gases, such as H_2O, CO, CO_2) requires a much higher energy. Therefore, the species relevant here must be relatively large, which would certainly prevent them from physically diffusing into the crystal lattice. Indeed, it has been recently shown through detailed SIMS, XPS and AFM study that even small molecular gases, such as O_2, cannot permeate rubrene lattice, unless the surface is forcefully photooxidized under a bright light with a formation of surface oxide defects [5]. Thus, it is very unlikely that the gauge species can physically penetrate into the crystal.

(3). We have performed a control experiment, in which rubrene crystals were coated with a self assembled monolayer (SAM) of fluoro-alkyl-trichlorosilane (FTS), placed in a vacuum chamber and exposed to a high-vacuum gauge. Rubrene-FTS interface is conducting (see, e.g., [[12]]) and can be monitored *in-situ* in high vacuum as a function of high-vacuum gauge operation. We have done these measurements and observed that gauges had absolutely no impact on conductivity of rubrene-FTS interface. The surface of the crystal coated with only a 1.3 nm-thick monolayer (FTS SAM) had virtually no response to the gauge, irrespectively of the duration of the exposure or the vacuum level. Such behavior is in stark contrast with pristine uncoated rubrene. This control experiment suggests that a layer of nanoscale thickness (SAM), separating the crystal from the gauge radicals landing at the surface, is sufficient to protect the conducting surface from the gauge effect species, which points to a short-range nature of the gauge effect.

(4). Finally, we have carried out an additional control experiment, in which a pristine rubrene crystal with contacts was first coated with a thin layer of parylene (a conformal, pin-hole

free, transparent polymer insulator) and then placed in a high vacuum chamber. The parylene thickness was d = 280 nm (that is, much smaller than the typical light penetration length in rubrene in the visible range of spectrum, $\boldsymbol{\alpha}^{-1}$ ~ a few μm). Measurements of this sample under a white-light photoexcitation showed that there were absolutely no degradation of photoconductivity after the gauge was turned on (Fig. S1). Thus, a thin ($d \ll \boldsymbol{\alpha}^{-1}$) protective layer is sufficient to prevent the gauge effect from interfering with photocarriers, which once again suggests a short-range nature of the gauge effect.

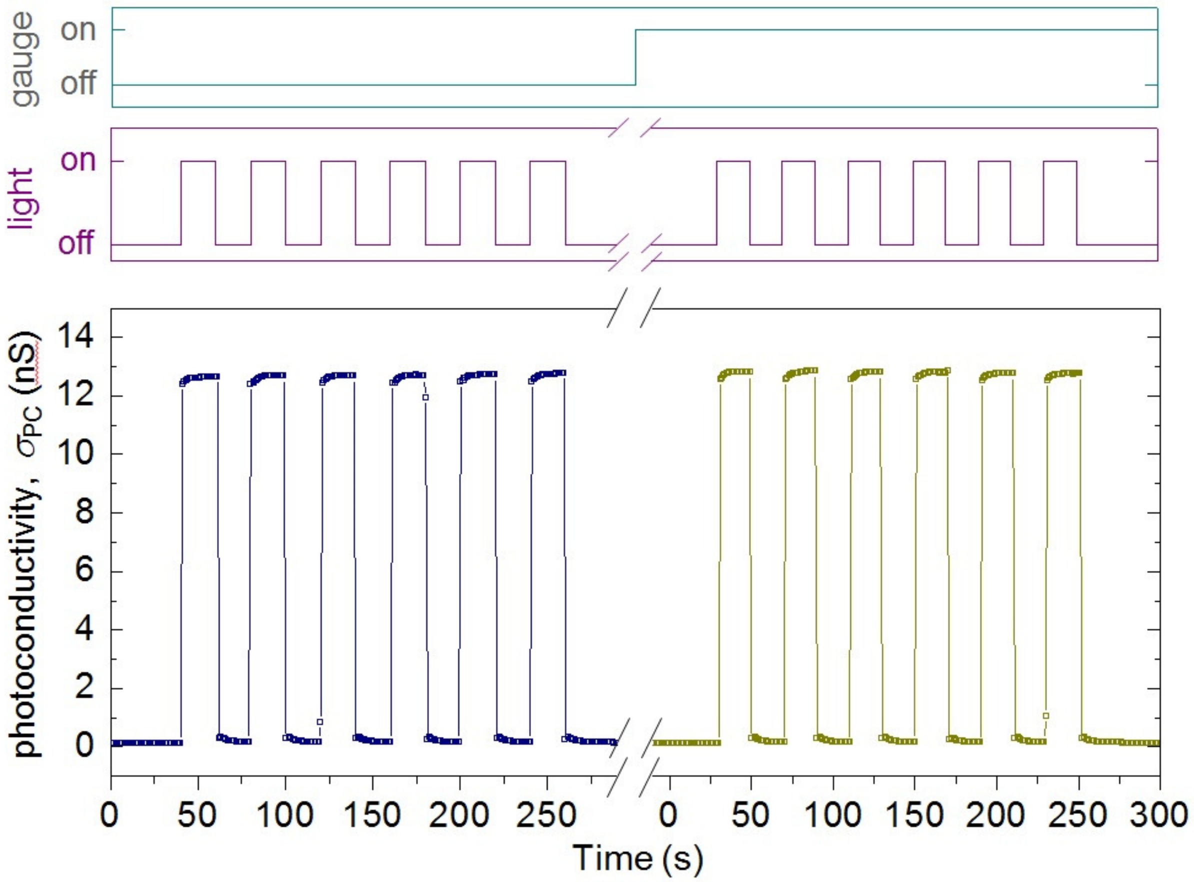

Fig. S1. Demonstration of a short-range nature of the gauge effect. Photoconductivity of a pristine rubrene crystal conformally coated with a thin (280 nm) layer of parylene before (left) and after (right) a high-vacuum gauge was turned on. Photoexcitation was achieved with a white light produced by a 20 W quartz-tungsten-halogen lamp. Light penetration length in rubrene in the visible range is $\boldsymbol{\alpha}^{-1} > 1$ μm. Vacuum level: 10^{-5} Torr. It is evident that a protective layer much thinner than $\boldsymbol{\alpha}^{-1}$ completely eliminates the gauge-effect degradation of photoconductivity.

These experiments show that the only mechanism by which the gauge-effect species could influence the charge transport is trapping of mobile carriers at the surface of the crystal (generation of surface traps).

An additional advantage of using gauge effect is that the transition from a surface to a bulk mode is reversible, because the gauge effect can be fully recovered within a few hours by exposing the damaged samples to air or oxygen atmosphere under illumination (for details, see [8]). Despite free radicals being rather reactive species, such recovery is possible due to the powerful mechanism of free radical scavenging by singlet molecular oxygen (which is the first excited state of O_2 molecule) known in spin-selective radical chemistry [13]. This occurs via a triplet-to-singlet conversion of O_2 in a vicinity of rubrene molecules (at the surface of the crystals) through the photosensitized energy transfer from excited rubrene to O_2 [4]. Thus, we can intentionally switch between the two distinct regimes of photoconductivity of the same sample and measure the photoexcitation intensity dependence, $\sigma_{PC}(G)$, of either the surface or the bulk photocurrents independently.

Gauge effect should not significantly influence the triplet exciton dissociation rate at the surface, because the typical density of traps generated by high-vacuum gauges at the surface (10^9 - 10^{12} cm^{-2}) is much smaller than the density of rubrene molecules comprising the (***a***,***b***) facet of the crystal (10^{14} cm^{-2}) [8]. Such a small trap density is inferred from the measurements of the threshold voltage shift in vacuum-gap OFETs under typical gauge effect exposure conditions. Thus, a triplet exciton diffusing toward the top (***a***,***b***) surface of the crystal from the bulk would encounter a pristine rubrene molecular site with a much higher probability than it would encounter a surface trap. Therefore, only a negligible fraction of triplet excitons arriving to the top surface would interact with such a trap first, making any possible outcome of such an interaction statistically insignificant. Hence, the overall triplet dissociation rate (averaged over macroscopically large surface of the sample) should not be significantly affected by the gauge species sparsely distributed over the surface.

3. Photoconductivity and intentional (gradual) photooxidation of rubrene crystals.

We have shown in the main text that the excitation density dependence of photoconductivity, $\sigma_{PC}(G)$, in the bulk and surface regimes in rubrene crystals are remarkably different. For the most commonly used photoexcitation intensities, the surface photoconductivity follows a power law, $\sigma_{PC}(G) \propto G^{\alpha}$, with the exponent $\alpha = 1/3$, while the bulk photoconductivity is described by another exponent $\alpha = ½$ (note: this exponent α is not to be confused with the absorption coefficient $\boldsymbol{\alpha}$). We would like to additionally demonstrate that, in principle, all the intermediate states of photoconductivity, between the two limiting regimes of a pure surface (in pristine crystals) and pure bulk (in gauge-effect treated crystals) photoconductivities are possible in trap-dominated crystals. To show this, we intentionally introduced traps in pristine crystals by photooxidation under white light in O_2 atmosphere, similar to the technique recently used by Najafov *et al.* [14]. Exposing the crystal to doses of illumination in oxygen or air results in a gradual (and thus controlled) surface oxidation with increasing density of traps. This has been shown to systematically reduce the dark and photoconductivity of rubrene crystals [14]. In the experiment presented in Fig. S2 below, a pristine rubrene crystal was first measured and then exposed to a bright white light (100 $mW\cdot cm^{-2}$) in air for a short period of time (~ 10 s), which has immediately led to a reduction of the dark and photoconductivity of the sample. After each additional dose of photooxidation, dark and photoconductivity were measured in high vacuum (with the gauge off). The resultant set of $\sigma_{PC}(G)$ curves reveals a remarkable result (Fig. S2). Each dose of photooxidation reduces the absolute value of photoconductivity and leads to a gradual evolution from the power exponent $\alpha = 1/3$ (corresponding to the surface-dominated photoconductivity in pristine crystals) to $\alpha = ½$ that corresponds to pure bulk photoconductivity. Interestingly, the drastic reduction of the absolute value of photoconductivity in this process (by almost two orders of magnitude) is irreversible, contrary to the case of the gauge-effect treated samples that can be recovered. However, the final state corresponding to fully oxidized surface (the lowest curve in Fig. S2) is identical in behavior to the gauge-effect treated samples that also show $\sigma_{PC} \propto G^{0.5}$ dependence with $\alpha = ½$ and a very small dark conductivity, σ_0 ~ 50 pS.

This additional experiment shows that the exact method of introducing surface defects is not important: we observe a similar transformation from $\alpha = 1/3$ to $\alpha = ½$ (accompanied by a drastic reduction of the absolute value of photoconductivity) after exposing the crystals to high-vacuum

gauges, intentionally introducing microscopic mechanical scratches at the (***a***,***b***) facet between the contacts, as well as photooxidation.

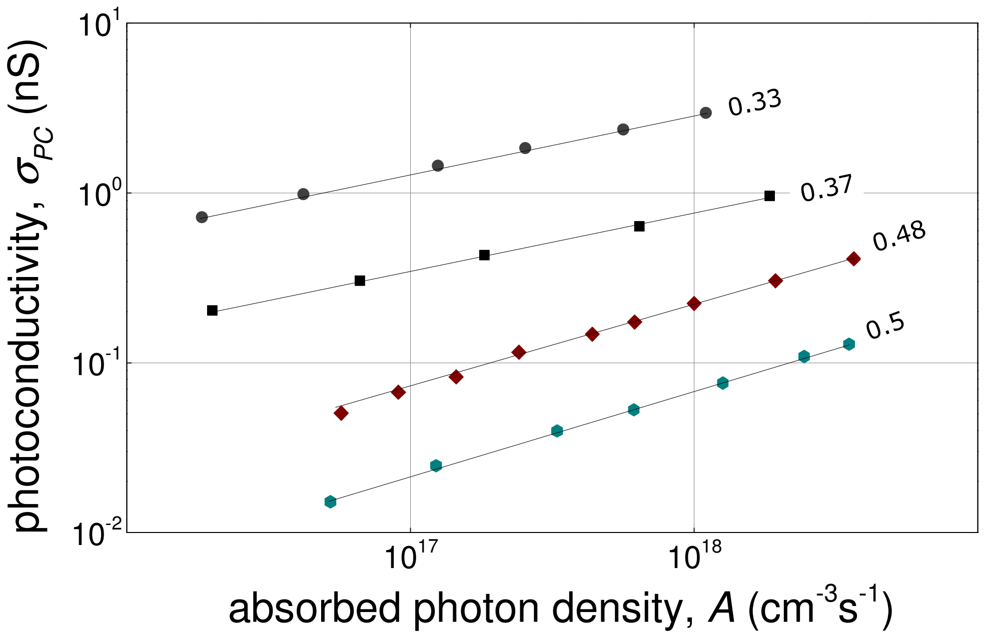

Fig. S2. Excitation density dependence of photoconductivity in a gradually (photo)oxidized rubrene crystal. For ease of comparison, only the intermediate range of excitation densities is shown. Photooxidation was performed by exposing the crystal to short doses of illumination in air, as described in sec. 3 of this Supplementary Information. Photoexcitation for σ_{PC} measurements was achieved by illuminating the (***a***,***b***) facet of the crystal at a normal incidence with a monochromatic light (λ = 500 nm, penetration length $\boldsymbol{\alpha}^{-1}$ = 1 μm) in high vacuum with the gauge off. As the density of oxygen-related traps near the surface of the crystal increases, $\sigma_{PC}(G) \propto G^{\alpha}$ dependence clearly shows a gradual transformation from α = 1/3 (surface photoconductivity) to α = ½ (bulk photoconductivity).

4. Comparison of the mechanisms of sub-linear photoconductivity in *rubrene* and *Si*.

It can be seen in Fig. S3a that the photocurrent response of rubrene crystals becomes sublinear already at very low excitation powers. Sublinearities are sometimes observed in organic or inorganic semiconductors and are often attributed to either an electron-hole (*np*) recombination (for α = 1/2) or the cooperation of traps and recombination centers (for $\alpha \neq 1/2$). Sublinearity with α lower than ½ at elevated temperatures was modeled for an organic

semiconductor in Ref. [15] assuming the diffusion dominant transport of photoexcited carriers and an exponential trap distribution. $\alpha = 1/3$ dependence at high illumination intensities observed in crystalline germanium can be expected when Auger recombination (that is, a non-radiative, three particle recombination, *nnp* or *ppn*) is dominant, and carrier diffusion is negligible [16].

Given the recent progress in the growth of organic single crystals and the availability of samples with unprecedented chemical purity (such as rubrene), we do not expect that residual small concentration of traps in pristine crystals can be sufficient to result in the observed sublinearities in σ_{PC}.

We propose that when the equilibrium concentrations of excitons and charge carriers in our *cw* measurements become sufficiently high, an additional loss mechanism, given by the triplet-charge quenching term ($T \cdot n$) in the rate equations 1-3 formulated in the main text, becomes not only relevant, but dominant. As we show in the main text, this can ultimately lead to $\alpha = 1/3$ power dependence of photoconductivity. It must be noted that a regime with $\alpha = 1/3$ can, in principle, result from a third-order Auger carrier recombination. Such processes can occur, typically in indirect-gap semiconductors (for instance, Si or Ge), where radiative recombination of electrons and holes is forbidden, and a third carrier is necessary in order to carry away the excess energy and momentum [17,18,19]. This can typically be observed in Si or Ge at a very high concentration of photocarriers.

Indeed, as we show in Fig. S3b for crystalline Si(111), one can achieve a situation when a third-order (three-particle) mechanism of sublinearity in photoconductivity becomes apparent. This can only be observed when using a very high photoexcitation intensity (when the photocarrier concentration is high), or when the material is of exceptionally high purity and has a high carrier mobility, that is, when other recombination channels (involving bang-gap states) are suppressed. This third-order recombination mechanism is related to the Auger effect, in which the excess energy and momentum are transferred to a third carrier that gets excited to a higher energy level within the band. After such a three-particle interaction, the third carrier eventually losses its excess energy to thermal lattice vibrations.

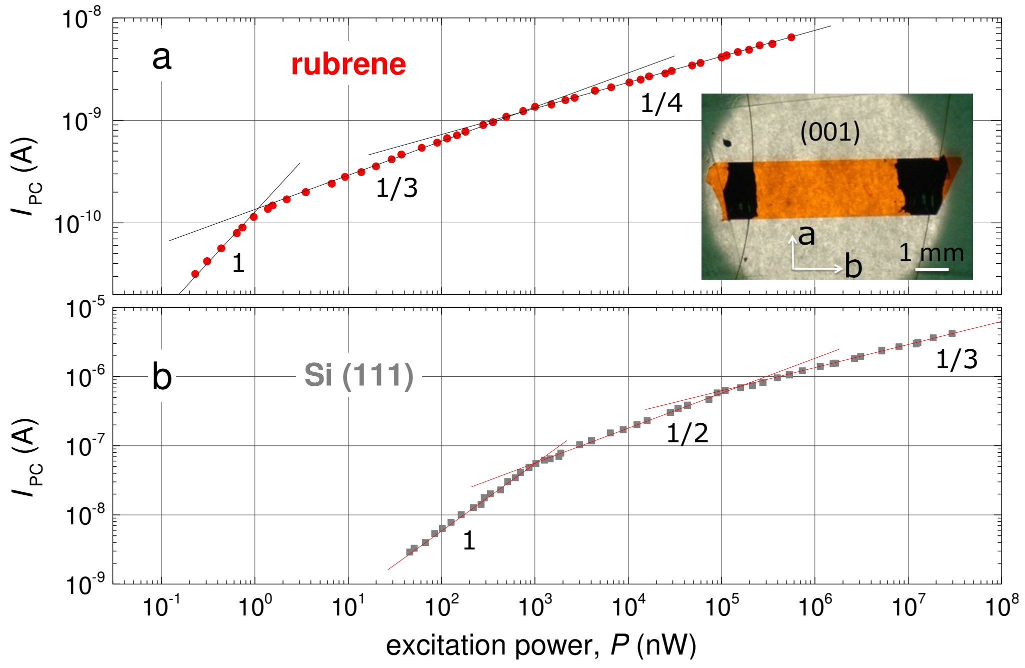

Fig. S3. Excitation density dependence of photoconductivity in (a) pristine rubrene crystals, compared to that in (b) single-crystal Si(111). (a): Photoexcitation achieved by illuminating the (***a***,***b***) facet of the rubrene crystal at a normal incidence with a monochromatic light (λ = 500 nm, $\boldsymbol{\alpha}^{-1}$ = 1 μm) in high vacuum with the gauge off and V = 1 V applied between the contacts separated by 4 mm (see main text for sample geometry). **(b):** Photoexcitation achieved by illuminating the (111) facet of the high-resistivity Si sample (ρ = 30 kΩ cm) at a normal incidence with a monochromatic *cw* laser light (λ = 532 nm, $\boldsymbol{\alpha}^{-1}$ = 1.36 μm) in high vacuum with the gauge off and V = 1 V applied between contacts separated by 5 mm. All experimental conditions, including the sample dimensions and excitation light penetration lengths, were intentionally chosen to be very similar for convenient direct comparison between the two materials. Note that the deviation from the linear growth in the case of pristine rubrene occurs at many orders of magnitude lower excitation power compared to Si, which suggests that third-order Auger recombination mechanism is an unlikely mechanism for rubrene, and a model based on a second-order $T{\cdot}n$ annihilation should be favored (see main text).

Our experiments show that in rubrene single crystals, the onset of power-law dependence with α = 1/3 occurs at a very low excitation intensity (5-6 orders of magnitude lower than that in Si), and this regime persists over a wide range of excitation powers (Fig. S3). One must consider the difference between these two materials with respect to the particle interactions and dynamics. While in Si (at room temperature) photon absorption leads to a direct creation of mobile electrons and holes, photon absorption in rubrene leads to a generation of excitonic states with

relatively long lifetimes of a few nanoseconds for singlets and ~ 100 microseconds for triplets, and charge carrier generation in rubrene is a secondary process that occurs due to assisted dissociation of such long-lived excitons. This results in a situation when steady-state photoexcitation can produce relatively large populations of triplet excitons and charge carriers, and new decay channels, such as triplet-charge quenching, become dominant. This, however, is not expected in Si because of the absence of stable excitons at room temperature.

The fact that sublinearities in the power dependence of photoconductivity in Si occur at much greater excitation densities than in rubrene (Fig. S3), when the two systems are measured in similar experimental conditions, is consistent with the bulk vs. surface nature of photoconductivity in Si and pristine rubrene, respectively. In addition, the sequence of exponents in pristine rubrene is different from that in Si: in pristine rubrene (surface photoconductivity) we have $\alpha = 1$, then 1/3, and then ¼, with the electron-hole recombination regime ($\alpha = 1/2$) missing. This indicates that $\alpha = 1/3$ regime in rubrene cannot be assigned to a three-particle Auger recombination, as in such a case it would have been preceded by the $\alpha = ½$ regime due to a more probable *np* recombination. This clearly shows that the mechanisms of sub-linearities in photoconductivity in molecular crystals are *fundamentally different* from those in inorganic semiconductors.

5. Refinement of the photoconductivity "oscillations" model (Ref. [7]).

The sub-linear excitation intensity dependence of photoconductivity observed in this work spans many orders of magnitude in excitation powers and covers the range typically used in laboratory studies of photophysical properties of organic materials. For instance, the regime described by the function $\sigma \propto G^{1/3}$ (the so-called $\alpha = 1/3$ regime) extends over three-four orders of magnitude in illumination intensity (Figs. 1 and 3b of the main text and Fig. S3a above). Therefore, it is very likely that most (if not any) existing measurements, in which the excitation density is varied, are affected by this dependence.

An important measurement that has been recently performed in rubrene concerns the modulations of photoconductivity with the polarization angle of light, $\sigma_{PC}(\theta)$ (the so-called "oscillations" in photoconductivity) [7]. Here, θ refers to an angle between the polarization of a

monochromatic linearly polarized light normally incident on the (***a***,***b***) facet of rubrene crystal and the crystallographic ***b***-axis of the crystal (the high-mobility axis). As usual, the photoconductivity in this experiment has been measured in a co-planar contact geometry at the (***a***,***b***) facet of the crystal. It has been found that in high-purity pristine rubrene crystals, the contrast of photoconductivity modulations, η_σ, is much smaller than the corresponding contrast of the absorption coefficient, $\eta_{\boldsymbol{\alpha}}$ (Fig. S4 below, reproduced from Ref. [7]). Here, again, the absorption coefficient of rubrene, $\boldsymbol{\alpha}$, should not be confused with the power exponent α in σ(*G*) dependence. The contrasts in σ_{PC} and $\boldsymbol{\alpha}$ are defined as:

$$\eta_\sigma \equiv (\sigma_{\mathbf{b}} - \sigma_{\mathbf{a}})/\sigma_{\mathbf{b}} \qquad \text{and} \qquad \eta_{\boldsymbol{\alpha}} \equiv (\alpha_{\mathbf{b}} - \alpha_{\mathbf{a}})/\alpha_{\mathbf{b}}, \qquad \text{respectively.}$$

For example, in pristine rubrene crystals, the range of σ-contrast values obtained in a large number of samples at 495-nm excitation is η_σ = 3 - 12 %, with more typical values of 5 - 9 % observed in high-purity crystals, while the corresponding modulations of the absorption coefficient $\boldsymbol{\alpha}(\theta)$ are much greater, $\eta_{\boldsymbol{\alpha}}$ = 42 % (Fig. S4, see also Ref. [7]).

This effect can only be rationalized by assuming a long-range diffusion of photogenerated excitons to the surface of the crystal, where they dissociate and generate surface photocurrent. In this model, photon absorption initially results in a *Beer-Lambert* distribution of singlet excitons below the (***a***,***b***) surface of the crystal, shown as a hatched area in Fig. S4b. Singlet excitons in rubrene rapidly undergo fission into triplets that are forbidden from a radiative recombination and therefore have a long lifetime (τ_T ~ 100 μs) [2]. Singlet fission in rubrene crystals has been confirmed in several recent experiments (see refs. [9], [12], [23], [24] and [25] of the main text). The rather low integral *PL* quantum yield in rubrene crystals (only about 1-2 % at low to moderate photoexcitation intensities) is consistent with singlet fission occurring in this material. Such *PL* quantum efficiency is still greater than that in tetracene or pentacene, which are both known to be singlet fission materials. This however can be understood based on the fact that in tetracene and pentacene, the first excited triplet energy T_1 is smaller than one half of the singlet energy S_1, and thus triplet fusion requires a thermal activation. On the contrary, in rubrene, triplet energy is believed to be very close to one half of the singlet energy, and thus fusion process is rather efficient at room temperature, leading to a recovery of some singlets. This makes *PL* quantum yield in rubrene higher in *cw* measurements. The triplet excitons in rubrene

can migrate throughout the crystal over the distances much greater than the typical diffusion range for singlet excitons and dissociate on defects (including the natural boundaries of crystals – their surfaces), thus generating photoconductivity.

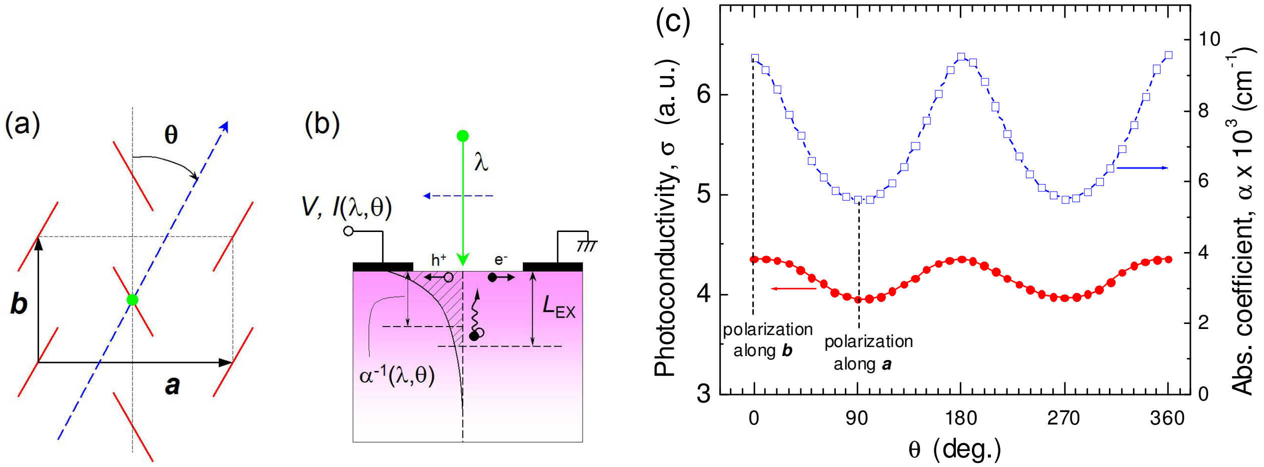

Fig. S4 (reproduced from Ref. [7]). (a) Molecular packing in the (***a***,***b***) plane of rubrene: long molecular axes are shown by the red segments, blue dashed arrow defines the polarization of a normally incident light with an angle θ with respect to the ***b***-axis. (b) A schematic "side view" of the device showing electrical contacts at the (***a***,***b***)-facet of the crystal probing the photocurrent response of the sample, $I(\lambda,\theta)$, to incident light (green arrow). An exponential profile of the density of photogenerated excitons in the crystal defined by the light penetration length, $\alpha^{-1}(\lambda,\theta)$, and an exciton diffusion range, L_{EX}, are shown. (c) Experimental dependence of the photocurrent on polarization angle for a 495-nm excitation (solid red circles), measured in a pristine rubrene crystal with thickness d = 1 mm >> α^{-1}(495 nm) ≈ 1 μm (that is, absorbing all the incident photons). Angular dependence of the absorption coefficient, $\alpha(\theta)$, for 495-nm excitation in rubrene (open blue squares).

This effect has been modeled by Najafov *et al.* by taking the integral that counts the total number of triplet excitons reaching the top surface of the crystal (for details, see Ref. [7]). This integral is reproduced here:

$$\sigma_{PC}(\theta, \lambda) = \sigma_{surf} = \gamma_0 \chi_0 \Phi_0 \alpha \int_0^\infty exp(-\alpha \cdot x) \cdot exp(-x/L_{EX}) \cdot dx = \gamma_0 \chi_0 \Phi_0 \cdot \frac{\alpha L_{EX}}{\alpha L_{EX} + 1} \qquad \text{(S1)}$$

In this integral, $\alpha \cdot \Phi_0 \cdot \exp(-\alpha x) \cdot dx$ is the number of photons absorbed in a layer of the crystal below the (***a***,***b***) surface from x to x + dx (x represents the depth into the crystal with x = 0 corresponding to the top (***a***,***b***) surface of the crystal). The second exponent, $\exp(-x/L_{EX})$, is

related to the probability for a triplet exciton generated at the depth x to reach the top surface of the crystal. According to this model, the photoconductivity oscillations would then be described by the contrast:

$$\eta_\sigma \equiv \frac{\sigma_b - \sigma_a}{\sigma_b} = 1 - \frac{\alpha_a}{\alpha_b} \cdot \frac{\alpha_b L_{EX} + 1}{\alpha_a L_{EX} + 1} \quad \text{(S2)}$$

In this equation, the triplet exciton diffusion length, L_{EX}, is the only fitting parameter, with $\boldsymbol{\alpha}_{\mathbf{a,b}}(\lambda)$ being the experimental absorption coefficients measured for polarizations along ***a*** and ***b*** axes of the crystal.

In light of the new knowledge of nonlinear surface photoconductivity described in the current work (see the main text) the above model of Najafov *et al.* must be adjusted. According to the model of the regime with power exponent $\alpha = 1/3$ (this regime occurs at most commonly used photoexcitation intensities), photoconductivity in this regime can be derived from the set of rate equations given in the main text (eqs. 1-3) under the assumption that the triplet fusion term in eqs. 1 and 2 (proportional to T^2) is not yet dominant, the two other terms (T/τ_T and βT) are also small and can be neglected, because the triplet lifetime τ_T is long, and the quantum efficiency of triplet dissociation at the surface, β, is relatively small. This leaves only two dominant terms in eq. 2: the triplet generation via singlet fission ($2f_S S/\tau_S$) and the triplet-charge quenching at the surface ($\gamma_a T\cdot n$). Thus, in this regime, the set of rate equations 1-3 reduces to the following:

$$\frac{dS}{dt} = G - \frac{S}{\tau_S} \quad \text{(S3)}$$

$$\frac{dT}{dt} = 2f_S \frac{S}{\tau_S} - \gamma_a T n \quad \text{(S4)}$$

$$\frac{dn}{dt} = \beta T - \gamma_n n^2 \quad \text{(S5)}$$

These equations lead to the following steady-state ($dS/dt = dT/dt = dn/dt = 0$) solution for the densities of singlets, triplets and photocarriers: $S \propto G$, $T\cdot n \propto G$, and $n \propto G^{1/3}$.

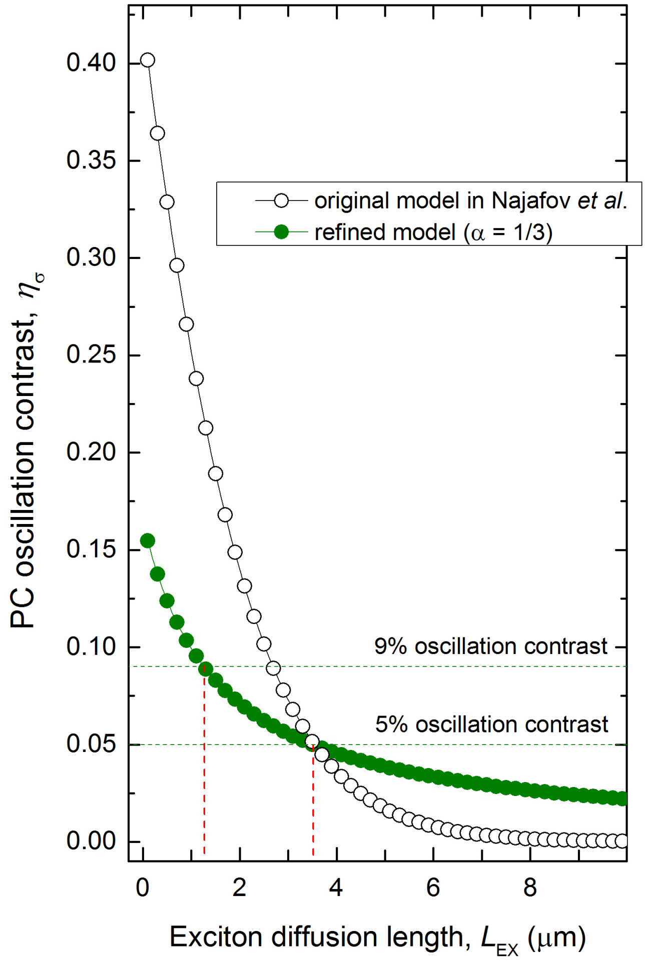

Fig. S5. The contrast of photocurrent modulations, η_σ, plotted as a function of exciton diffusion length, L_{EX}, calculated by Najafov et al. [7] (black open symbols), and that calculated by using the refined Eq. S8 that accounts for the sublinear photoconductivity in the regime $\sigma_{PC} \propto G^{1/3}$ (green solid symbols). Both curves were obtained using the experimental values of the absorption coefficient α for 495-nm excitation polarized along ***b*** and ***a*** axes of rubrene (α_a = 5.5×10^3 cm^{-1}, and α_b = 9.5×10^3 cm^{-1}). The horizontal dashed lines mark the range of typical experimental values for the oscillation contrast in σ, η_σ = 5 -9 %. It can be seen that in the refined model, the exciton diffusion length is quite substantial, L_{EX} > 1 μm.

Note that eq. S4 for the steady-state density of triplets T has two terms: 1st is the source of triplets via singlet fission, and 2nd is the most relevant (in this regime) channel of triplet decay via triplet-charge quenching. The combination of these competing processes is what determines the steady-state triplet population, T, which in turn governs the photocarrier production described by eq. S5. The source of triplets via singlet fission plays an equivalent role as the integral used

by Najafov *et al.* in their original modeling of photocurrent oscillations (the integral in eq. S1 above) [7]. The only difference between that integral and the term $2f_S S/\tau_S$ in eq. S4 is that the integral calculates the portion of triplets that reach the top surface of the crystal via diffusion characterized by exciton diffusion length, L_{EX}, while the 1st term in eq. S4 refers to the overall triplet population.

Thus, in order to make the necessary refinement of Najafov's model, one has to take into account both the triplet source and the triplet decay channels, so that a steady-state population of triplets, *T*, is used, instead of just the source of triplets. Such an adjustment of the model can be easily done by taking the expression on the right-hand side of eq. S1 (the result of the integration) and using it as the source of triplets in eq. S4:

$$2f_S S/\tau_S \quad \Rightarrow \quad \gamma_0 \chi_0 \Phi_0 \cdot \frac{\alpha L_{EX}}{\alpha L_{EX} + 1} \tag{S6}$$

After this, instead of $T \cdot n \propto G$ and $n \propto G^{1/3}$, we get:

$T \cdot n \propto \frac{\alpha L_{EX}}{\alpha L_{EX} + 1}$ (from eq. S4), and $n \propto \left(\frac{\alpha L_{EX}}{\alpha L_{EX} + 1}\right)^{1/3}$ (from eq. S5), respectively, leading to the photoconductivity,

$$\sigma_{PC} \equiv en\mu \propto \left(\frac{\alpha L_{EX}}{\alpha L_{EX} + 1}\right)^{1/3} \tag{S7}$$

The refined expression for the contrast of photoconductivity oscillations will then take a form:

$$\eta_\sigma \equiv \frac{\sigma_b - \sigma_a}{\sigma_b} = 1 - \left(\frac{\alpha_a}{\alpha_b} \cdot \frac{\alpha_b L_{EX} + 1}{\alpha_a L_{EX} + 1}\right)^{1/3} \tag{S8}$$

This expression (instead of the original one in (S2)) should be used to model the photoconductivity oscillations. Figure S5 performs such a modeling using both the old formula from Najafov *et al.* [7] (eq. S2) and the new formula (eq. S8). The refined model (green solid symbols) leads to somewhat smaller exciton diffusion lengths for the oscillation contrasts > 5%, while it gives longer L_{EX} for smaller contrasts. Nevertheless, even at a very large oscillation contrast of 10 % (which is typically a sign of insufficiently purified crystals), the exciton diffusion length predicted by the refined model is still large, ~ 1 μm.

Finally, we would like to emphasize that the co-planar device geometry (electrical contacts separated by a large distance of 1-3 mm, deposited at the top surface of the crystal), as well as macroscopically thick crystals used in Najafov *et al*. [7] and in this work by Irkhin *et al*. ensure that there are no experimental artifacts related to the unaccounted effects of interference and exciton quenching at metal/organic interfaces that are very common in thickness-dependent PL quenching and photo-current excitation spectroscopy measurements in sandwiched thin films. An excellent review of various artifacts that can lead to erroneous measurements of exciton diffusion length in thin-film devices can be found in the recent paper by R. R. Lunt *et al*. [[20]].

References:

[1] R. W. I. de Boer, M. E. Gershenson, A. F. Morpurgo, and V. Podzorov, *Phys. Status Solidi* A **201**, 1302 (2004).

[2] P. Irkhin, A. Ryasnyanskiy, M. Koehler, and I. Biaggio, *Phys. Rev*. B **86**, 085143 (2012).

[3] V. Podzorov, V. M. Pudalov, and M. E. Gershenson, *Appl. Phys. Lett*. **85**, 6039 (2004).

[4] V. Nardello, M.-J. Marti, C. Pierlot, and J.-M. Aubry, *J. of Chem. Education* **76**, 1285 (1999).

[5] D. D. T. Mastrogiovanni, J. Mayer, A. S. Wan, A. Vishnyakov, A. V. Neimark, V. Podzorov, L. C. Feldman, and E. Garfunkel, *Sci. Reports* **4,** 4753 (2014).

[6] Y. Wakabayashi, J. Takeya, and T. Kimura, *Phys. Rev. Lett*. **104**, 066103 (2010).

[7] H. Najafov, B. Lee, Q. Zhou, L. C. Feldman, and V. Podzorov, *Nature Mater*. **9**, 938 (2010).

[8] V. Podzorov, E. Menard, S. Pereversev, B. Yakshinsky, T. Madey, J. A. Rogers, and M. E. Gershenson, *Appl. Phys. Lett*. **87**, 093505 (2005).

[9] http://www.chemguide.co.uk/organicprops/alkanes/cracking.html

[10] http://www.cem.msu.edu/~reusch/VirtualText/nonionic.htm

[11] J. Fossey, Free Radicals in Organic Chemistry, Wiley, New York (1995).

[12] M. F. Calhoun *et al*., *Nature Mater*. **7**, 84 (2008).

[13] A. L. Buchachenko, *Pure Appl. Chem*. **72**, 2243 (2000).

[14] H. Najafov, D. Mastrogiovanni, E. Garfunkel, L. C. Feldman, and V. Podzorov, *Adv. Mater.* **23**, 981 (2011).

[15] Y. Aoyagi, K. Masuda, and S. Namba, *J. Appl. Phys*., **43**, 249 (1972).

[16] R. E. Wagner and A. Mandelis, *Semicond. Sci. Technol*. **11**, 300 (1996).

[17] M. J. Kerr and A. Cuevas, *J. Appl. Phys*. **91**, 2473 (2002).

[18] A. Richter, S. W. Glunz, F. Werner, J. Schmidt, and A. Cuevas, *Phys. Rev*. B **86,** 165202 (2012).

[19] E. Yablonovitch and T. Gmitter, *Appl. Phys. Lett*. **49**, 587 (1986).

[20] R. R. Lunt, S. R. Forrest *et al*. *J. Appl. Phys.* **105**, 053711 (2009).
